# Magnetomechanical Properties of Fe-Si-B and Fe-Co-Si-B Metallic Glasses by Various Annealing Temperatures for Actuation Applications

**DOI:** 10.3390/s23010299

**Published:** 2022-12-28

**Authors:** Yu Sun, Xu Zhang, Sheng Wu, Xin Zhuang, Bin Yan, Wanhua Zhu, Christophe Dolabdjian, Guangyou Fang

**Affiliations:** 1 Aerospace Information Research Institute, Key Laboratory of Electromagnetic Radiation and Sensing Technology, Chinese Academy of Sciences, Beijing 100094, China; 2 School of Electronic Electrical and Communication Engineering, University of Chinese Academy of Sciences, Beijing 100049, China; 3 School of Physical Science and Technology, Inner Mongolia University, Hohhot 010021, China; 4 Yantai Research Institute of Harbin Engineering University, Harbin Engineering University, Harbin 264006, China; 5 Normandie Univ, UNICAEN, ENSICAEN, CNRS, GREYC, Bd Maréchal Juin, 14000 Caen, France

**Keywords:** Fe-based amorphous alloys, power efficiency, magnetoelectric (ME) effects

## Abstract

Fe-based amorphous alloys have advantages of low iron loss and high effective permeability, which are widely used in sensors and actuators. Power efficiency is one of the most important indicators among power conversion applications. We compared the magnetomechancial power conversion factors of metallic glassy ribbons FeCoSiB (Vitrovac 7600) and FeSiB (Metglas 2605SA1). We investigated the crystallization process under different annealing temperatures and tested the magnetomechancial coupling factors (k) and quality factors (Q) by using resonant and anti-resonant methods. We found that the maximum coupling factor of the annealed Vitrovac ribbons was 23% and the figure of merits k^2^Q was 4–7; however, the maximum coupling factor of the annealed Metglas ribbons was 73% and the maximum value of k^2^Q was 16. We can observe that the Metglas 2605SA1 ribbons have higher values of the magnetomechanical power efficiency than those of the Vitrovac 7600 ribbons, which means they are better to be used in subsequent research regarding acoustically driven antennas.

## 1. Introduction

Fe-based amorphous alloys have been wildly used in laminated magnetoelectric composites as magnetic sensors, acoustically driven antennas, and power conversion devices due to their good magnetic and magnetoelastic properties [1,2,3,4,5]. Magnetoelectric antennas, which produce low-frequency magnetic field signals, are important in a variety of applications. However, the realization of this low-frequency antenna technology is a worldwide problem, and there are many difficulties to overcome. For example, high internal losses in the antennas hinder the augmentation of performances. The Defense Advanced Research Projects Agency (DARPA) launched the AMEBA program in 2017 seeking to develop portable, small, lightweight, high-performance very low frequency (VLF, 3–30 kHz) and ultra-low frequency (ULF, 300–3000 Hz) transmitters on land, in water, and underground [6,7]. Existing small antennas are based on electromagnetic resonance, so the size of the antenna depends on the wavelength of the electromagnetic wave, and the practical application of antenna length is at least a tenth of the length of the wave. In the recent decade, further antenna miniaturization has been an existing problem [8,9,10,11]. The new ME antenna, the size of which is less than a thousandth of the wavelength, has been designed to achieve a reduction of 1–2 orders of magnitude over the most advanced small antennas with no performance degradation [12]. Acoustic excitation antenna technology is a major application demand for low-frequency antenna maneuvering and high-frequency antenna chipping. New mechanisms, new materials, and new processes of multi-band miniaturized acoustic excitation antenna have been studied to break through the technical bottleneck of antenna size reduction in orders of magnitude and the physical limit of radiation efficiency and the bandwidth of traditional antenna and realize the leap of antenna technology in size and performance. It can be seen that the application prospects of this kind of antenna are very considerable. Therefore, the technical research regarding acoustic excitation antenna based on Fe-based metallic glass has great economic significance and social benefits. In general, the coupling factor (k) qualifies part of the magnetic energy converted into mechanical energy and can be used to characterize the magnetomechancial properties in Fe-based amorphous alloys [13,14].

Since Duwez et al. successfully produced amorphous alloys by using a quenching process in the 1960s [15], many researchers have made substantial progress in this field. Yao et al. reported the historical perspective and future direction of Fe-based soft magnetic amorphous/nanocrystalline alloys [16]. It was concluded that Fe-based amorphous alloys are excellent magnetic materials that have the advantages of low coercivity, high effective permeability, and low iron loss values. Arai et al. measured the values of k in the amorphous alloy Fe_80_P_13_C_7_ annealed under different temperatures [17]. The remarkable value of k was found to be 0.53. Brouha et al. reported that field annealing on Fe_80_B_15_Si_5_ under 350 °C for 2 min can result in the maximum k value of 0.86 with a 60 A/m longitudinal bias field [18]. Modzelewski et al. investigated and compared the magnetomechanical couplings of Metglas 2605CO and Metglas 2605SC [19]. It was also shown that extremely high magnetomechanical coupling factors can be realized in Fe-based metallic glasses. The coupling factors (k) for the above Fe-based amorphous alloys are shown in Table 1. In this paper, we compared magnetoelastic properties between FeCoSiB (Vitrovac 7600) and FeSiB (Metglas 2605SA1) by means of a mechanical resonance method, measured the crystallization process by using X-ray diffraction techniques, and studied the relevance of the crystallization and the enhancement of the magnetomechancial power efficiency in Vitrovac 7600 and Metglas 2605SA1.

## 2. Materials and Methods

Metallic glassy ribbons FeCoSiB (Vitrovac 7600, Hanau, German) and FeSiB (Metglas 2605SA1) were chosen to carry out the experiments. These two kinds of amorphous metals have often been used in magneto-electric (ME) composite materials due to their good magnetic and magnetomechanical properties [20,21,22,23,24]. In order to characterize their properties for use in ME composite materials, Vitrovac and Metglas ribbons of 25 μm thicknesses were cut into rectangular shapes with a length of 30 mm and a width of 3 mm to perform the experiments.

**Figure 1:**
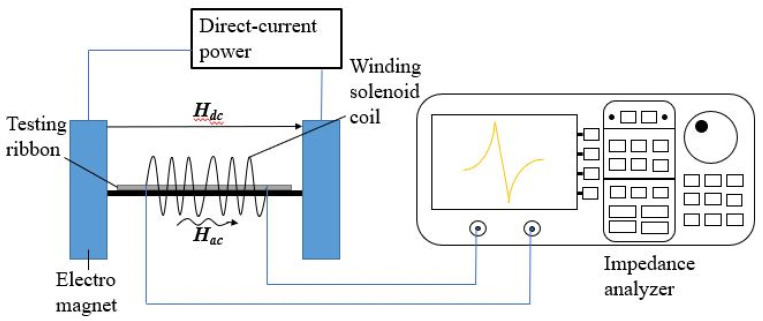
Schematic diagram of the experiment.

After annealing at different temperatures [25,26,27,28], the ribbons were inserted into a winding solenoid coil with dimensions of 30 mm length, 6 mm inner diameter, and 10 mm outside diameter with a 0.09 mm thick magnetic wire. This coil had an intrinsic inductance value of around 3 mH at 70 kHz. When the metallic glassy ribbons were driven by a magnetic excitation near the mechanical resonant frequency [29,30], the impedance and inductance values were extremely sensitive to the applied field to the sample [31,32]. Thus, we used the high-precision impedance analyzer (HP 4294A) to measure the resonant and anti-resonant frequencies on the motion impedance curve under an external magnetic bias field (H_dc_) [33]. The schematic diagram of the experiment is shown in Figure 1. The formula of the coupling factor can be derived from the motion impedance [34]. It can be by expressed by

(1) $$k^{2}=\frac{\pi^{2}}{8}\left(1-\frac{{f_{r}}^{2}}{{f_{a}}^{2}}\right)$$

where f_r_ and f_a_ are the resonance frequency and anti-resonant frequency on the impedance curve vs. frequency. We reduced the testing voltage applied on the input coil from 1 V to 100 mV until we found that the values of resonant/anti-resonant frequencies became constant.

The quality factor is another important parameter that usually defines the relationship between the power storage and the power loss [34]. This factor can be determined by the formula Q = f_r_/Δf, where Δf is the −3 dB bandwidth around the resonance. Specifically, Q can be directly measured considering the two poles on the inductance curve vs. frequency. It gives

(2) $$Q=\frac{f_{r}}{\Delta f}=\frac{f_{r}}{f_{2}-f_{1}},$$

where f_1_ and f_2_ are the frequency values for the maximum and minimum values of the inductance curve.

## 3. Results

**Figure 2:**
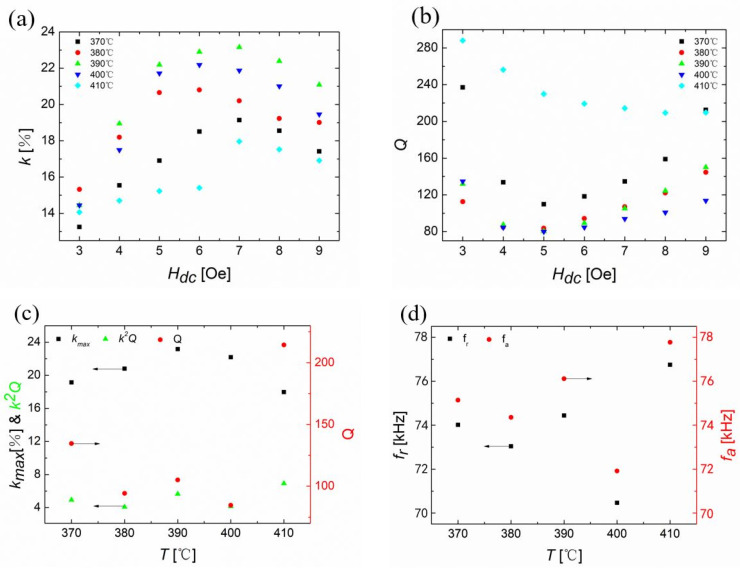
(**a**) The average value of the magnetomechanical coupling coefficient, kmean, for Vitrovac ribbons as a function of the bias field, H_dc_. Black, red, green, blue, and cyan curves represent the data for the ribbons annealed at 370 °C, 380 °C, 390 °C, 400 °C, and 410 °C for 20 min, respectively. (**b**) The average value of the quality factor, Q, of Vitrovac ribbons as a function of H_dc_. (**c**) The maximum values of coupling factors vs. H_dc_ and the associated quality factors, efficiency figures of merit, k^2^Q, as a function of the annealing temperature, T. (**d**) The resonance frequencies, f_r_, and anti-resonant frequencies, f_a_, associated with the maximum coupling factors, as a function of the annealing temperature.

The magnetomechancial coupling factors of Vitrovac ribbons were studied as a function of the magnetic bias field (H_dc_), as shown in Figure 2a. Five ribbons were annealed, respectively, under 370 °C, 380 °C, 390 °C, 400 °C, and 410 °C for 20 min and then cooled down to the room temperature in the air. For the annealed ribbon at 390 °C for 20 min, the maximum value of k was around 23% with an H_dc_ of 7 Oe (or 557 A/m) along the longitudinal direction of the ribbon. After that, the values of k decreased again when the Hdc increased to much higher values.

The quality factors of Vitrovac ribbons under different annealing conditions are shown in Figure 2b. For the ribbon that was annealed at 390 °C for 20 min, Q had a minimum value of around 80 at 5 Oe (or 398 A/m) H_dc_. When the annealing temperature increased, the required values of H_dc_ that could minimize Q increased. For example, the quality factors for the annealed ribbons under 410 °C needed an H_dc_ of 9 Oe (or 716 A/m) or even higher.

**Figure 3:**
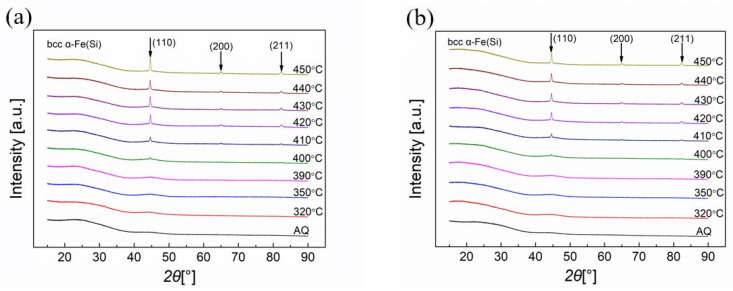
X-ray diffraction (XRD) patterns of Vitrovac ribbons on (**a**) shiny side and (**b**) dull side.

To study the crystallization process in annealed metallic glassy ribbons under different temperatures, the X-ray diffraction (XRD) patterns were taken from the shiny sides and dull sides of the Vitrovac ribbons, as shown in Figure 3a and Figure 3b, respectively. The XRD patterns were measured using Rigaku’s Smart Lab 9 kW diffractometer with Cu-target irradiation. Moreover, the one-dimensional array detector(D/tex) mode was used in our X-ray test, which has higher accuracy than the traditional scintillation counter (SC-70) mode. So, the broad XRD maximum at 25 degrees of 2 theta in Figure 3 most likely corresponded to the substrate [35,36]. The peaks of (110), (200), and (211) diffractive planes appeared on both sides of the ribbon when the annealing temperature exceeded 400 °C, and when the annealing temperature was higher, the crystallization degree became greater.

**Figure 4:**
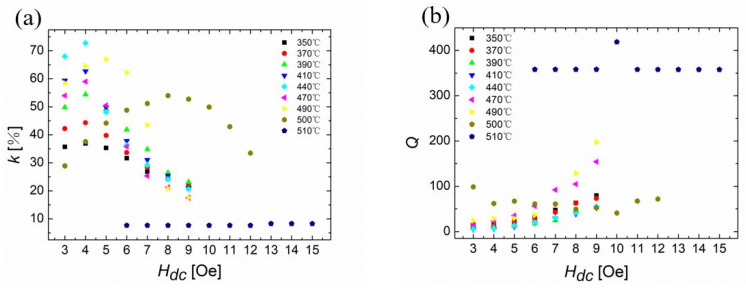

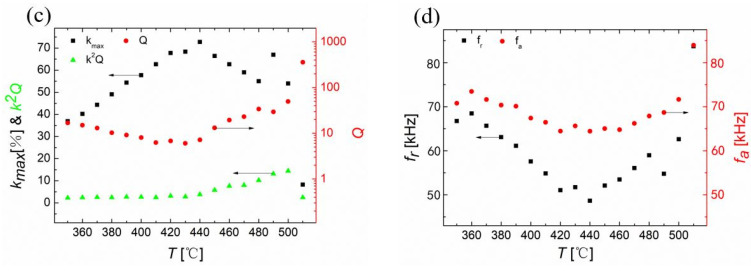
(**a**) The average value of the magnetomechanical coupling factors of Metglas 2605SA1 ribbons, k, as a function of the bias field, H_dc_. Black, red, green, blue, cyan, magenta, yellow, dark yellow, and navy curves are annealed at 350 °C, 370 °C, 390 °C, 410 °C, 440 °C, 470 °C, 490 °C, 500 °C, and 510 °C for 20 min, respectively. (**b**) The average value of the quality factors of Metglas ribbons, Q, as a function of the bias field, H_dc_. Black, red, green, blue, cyan, magenta, yellow, dark yellow, and navy curves are annealed at 350 °C, 370 °C, 390 °C, 410 °C, 440 °C, 470 °C, 490 °C, 500 °C, and 510 °C for 20 min, respectively. (**c**) The maximum value of coupling coefficient of Metglas ribbons, k, as a function of the annealing temperature, T. The green and red curves represent the values of the quality factors and efficiency coefficients corresponding to the maximum values of coupling coefficients, respectively. (**d**) The resonance frequencies, f_r_, and anti-resonant frequencies, f_a_, associated with the maximum coupling factors, as a function of the annealing temperature.

The values of k in Metglas ribbons were plotted as a function of H_dc_, as shown in Figure 4a. Ribbons were annealed under 350 °C to 510 °C with an interval of 10 °C and cooled down to the room temperature in air. The maximum values of k for the annealed ribbon at 440 °C for 20 min was around 73% with an H_dc_ of 4 Oe (or 318 A/m) along the longitudinal direction of the ribbon. However, the value of k decreased again when H_dc_ kept increasing. Moreover, when the annealing temperature exceeded 440 °C, the maximum value of k started to decrease. When the annealing temperature increased, the values of H_dc_ that could maximize k also increased. For example, to achieve the maximum k values, the annealed ribbons under 480 °C needed H_dc_ values of 4 Oe (or 318 A/m). However, the required value of H_dc_ for the annealed ribbon at 490 °C was 5 Oe (or 398 A/m) or even higher. The quality factors of Metglas ribbons under different annealing temperatures are shown in Figure 4b. For the ribbon that was annealed at 440 °C for 20 min, Q had a minimum value of around 6 for an H_dc_ of 3 Oe (or 239 A/m).

According to the reported results by Sagasti et al. in [37,38], when Metglas 2826MB3 strips (Fe_37_Ni_42_Mo_4_B_17_) were cut into rectangles with a length of 30 mm and a width of 3.33 mm, which is similar to the samples’ sizes in this paper, the maximum value of k was reported as a value of 26%, which was higher than that for Vitrovac ribbons but smaller than that for the Metglas ones. Moreover, the maximum values of the efficiency figure of the merit [39], k^2^Q, showed values of 7 for Virovac 7600, 16 for Metglas 2605SA1, and around 6.5 for Metglas 2826MB3.

**Figure 5:**
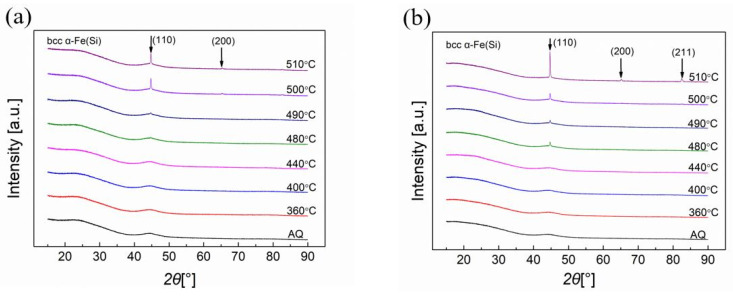
X-ray diffraction (XRD) patterns of Metglas ribbons on (**a**) shiny sides and (**b**) dull sides.

The X-ray diffraction (XRD) patterns of annealed Metglas ribbons on shiny sides and dull sides are shown in Figure 5a and Figure 5b, respectively. The intensity peaks of the diffractive planes (110), (200), and (211) for the body-centered cubic (bcc) α Fe(Si) crystallites appeared on both sides of the ribbon. This primary crystallization started to be observed when the annealing temperature increased to 490 °C on the shiny sides of the Metglas ribbons. However, the crystallization peaks were observed on the dull sides of the ribbons under the annealing temperature of 460 °C. This can be explained by the fact that the dull sides of the ribbons have lower fractions of metalloid elements than those on the shiny side [40,41,42].

**Figure 6:**
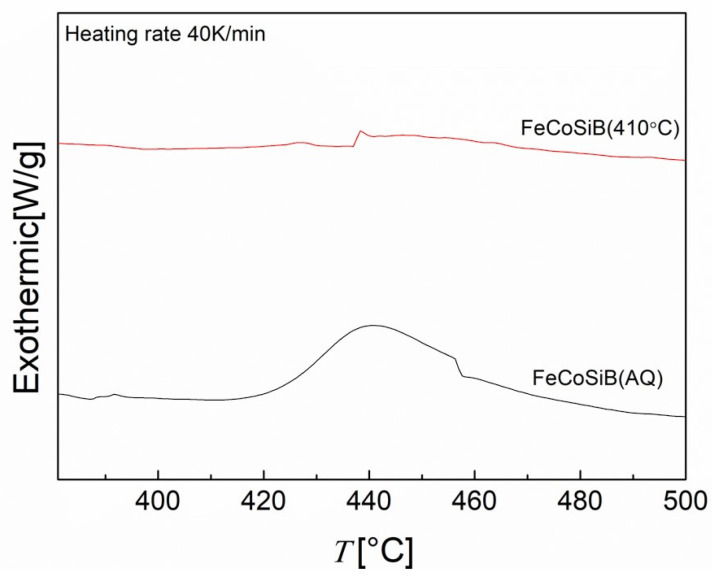
DSC curves of as-quenched (AQ) FeCoSiB and annealed FeCoSiB at 410 °C for 20 min.

Thermal stability was examined via differential scanning calorimetry (DSC) for AQ-FeCoSiB and the annealed FeCoSiB at a heating rate of 40 K/min, as shown in Figure 6.

**Figure 7:**
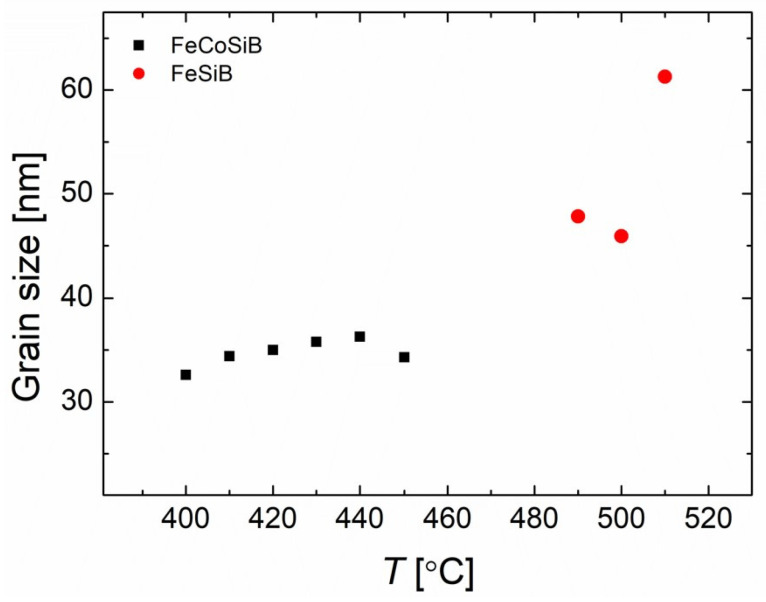
Grain size of FeCoSiB and FeSiB.

It was observed that the AQ FeCoSiB sample showed an obvious exothermic peak around 440 °C, which corresponded to the primary crystallization process from amorphous Fe to amorphous Fe + α Fe. However, the samples annealed at 410 °C for 20 min had already partially crystallized, so the exothermic peaks on the DSC curves were not so evident. This is consistent with the crystallization process of the samples with the XRD pattern. According to the study by G. Herzer and H. R. Hilzinger [43], the crystallization regions began from the surface of the ribbons with a grain size of several tens of nano meters to micrometers. This is consistent with our measurements. We found that the grain sizes in our experiments were also several tens of nano meters by using the Scherrer equation, as shown in Figure 7.

## 4. Discussion

We investigated the variation in the coupling coefficients and quality factors of two kinds of Fe-based metallic glasses, Vitrovac 7600 and Metglas 2605SA1, by performing different annealing conditions. We found that the annealed Vitrovac ribbons at 390 °C for 20 min showed the maximum coupling value of 23%. Additionally, the efficiency factors were values between 5 and 6. The annealed Metglas ribbons at 440 °C for 20 min could achieve a coupling factor of 73% with a longitudinal bias field of 4 Oe (or 318 A/m). However, the efficiency factor was only 4 with the bias of 4 Oe (or 318 A/m) in this case. When the annealing temperature was increased to 500 °C, the efficiency factor of Metglas ribbons increased to a value as high as 16.

Meanwhile, the crystallization under different annealing temperatures for Vitrovac and Metglas ribbons indicated that the annealing-induced partial crystallization could tune the magnetomechanical power efficiency factors. However, the effectiveness for tuning the efficiency was much more obvious in Metglas ribbons than that in Vitrovac ones. This was due to the fact that the intrinsic magnetomechanical coupling factor in Metglas 2605SA1 seemed much higher than that in Vitrovac 7600.

We observed that the traditionally annealed Metglas 2605SA1 only showed an efficiency factor of 4, which was less than that in Vitrovac 7600, but the annealed Metglas ribbons with partial crystallization exhibited an efficiency factor of 16. It was confirmed that the given Metglas ribbons with appropriate heat treatment are better for use in magnetostrictive actuators in terms of the magnetomechanical power efficiency [44,45], such as in magnetostrictive–piezoelectric–laminate-based acoustically driven antennas [4,12].

## Figures and Tables

**Table 1:** The magnetomechancial coupling factors of typical Fe-based amorphous alloys.

Amorphous Alloy Composition	Dimension (mm)	Coupling Factor kmax (%)	Annealing Temperature, Time T (°C), t (min)	Magnetic Bias Hdc (A/m)
Fe_73_Si_11_B_13_Nb_3_ (wire) [3]	3.9 (length), 0.1 (diameter)	43	345, 120	50
Fe_80_P_13_C_7_ (ribbon) [17]	60 × 2 × 0.03	53	350, 20	398
Fe_80_B_15_Si_5_ (ribbon) [18]	50 × 1 × 0.03	86	350, 2	60
Fe_67_Co_18_B_14_Si (ribbon) [19]	76 (length), 1.6 (width)	71	360–375, 10	835

## Data Availability

Data available on request due to restrictions.
